# Transparency of Geometric Distortions in Face Identity

**DOI:** 10.1068/ig4

**Published:** 2013-10-01

**Authors:** A Sandford, A M Burton, D White

**Affiliations:** University of Aberdeen, UK; University of Aberdeen, UK; University of New South Wales, Australia

## Abstract

Research has suggested that processing the spatial interrelationship of facial features is crucial to face perception (Diamond & Carey, 1986; Maurer, Le Grand, & Mondloch, 2002). However, recent research that has examined the effect of stretched and sheared images has suggested that early visual processes do not discriminate between an undistorted image and a severe geometric distortion of the same image (Bindemann, Burton, Leuthold, & Schweinberger, 2008). In two experiments, we show that both familiar and unfamiliar face recognition may be robust to geometric distortions. Using a familiar/unfamiliar judgment task, Experiment 1 compared correct reaction times and accuracy for undistorted face images to vertically and horizontally stretched front-view images. In Experiment 2, we compared undistorted face images to stretched, sheared, and twisted face images seen in front- and three-quarter-viewpoints. Observers saw all familiar and unfamiliar faces randomly in all distorted conditions. In both experiments, we found robust accuracy in all conditions despite effects on correct reaction times and substantial within-image deformation in distorted conditions. The findings suggest that processing the spatial interrelationship of features may not be crucial for correctly recognising a familiar face and correctly rejecting an unfamiliar face. This raises questions about the role of configural information in face processing for identity.

